# Effects of genotype and ecological environment on the community structure and function of symbiotic bacteria in rhizosphere of ginseng

**DOI:** 10.1186/s12866-022-02649-0

**Published:** 2022-10-03

**Authors:** Jiyue Zhang, Pin Liu, Botao Nie, Xinxin Liu, Zhen Zhang, Runlong He, Weiwei Dong, Wenxiu Ji

**Affiliations:** 1 College of Integration Science, Yanbian University, Yanji, Jilin Province 133002 China; 2 College of Agriculture, Yanbian University, Yanji, Jilin Province 133002 China

**Keywords:** Genotype, Ginseng, Growth environment, High-throughput sequencing, Microbial community, Rhizosphere

## Abstract

**Background:**

Ginseng, an important traditional Chinese medicine and a new resource food, has two production modes: farmland ginseng and forestland ginseng. Ginseng faces many problems such as high soil bulk density, easy hardening, low nutrient content, reduced porosity and increased soil acidification because of continuous cropping. Increasing studies indicate that plant rhizosphere symbiotic bacteria have an important effect on plant growth and development. We speculate that differences in microbial community may play an important role in promoting ginseng growth, development and health. To reveal the differences between farmland and forestland ginseng cultivation, and to address problems associated with continuous ginseng cropping, we investigated the effects of differences in plant rhizosphere symbiotic bacterial communities in promoting ginseng growth, development, and health.

**Result:**

In the present study, the microbial communities in the rhizosphere of different genotypes and ecological environments were analyzed using the high-throughput sequencing platform Illumina, phylogenetic investigation of communities by reconstruction of unobserved states (PICRUSt), and other technologies. The organic matter, total nitrogen, available nitrogen, and available phosphorus contents in forestland soil were significantly different from those in farmland. The bacterial communities of ginseng in forestland, farmland, and greenhouse environments have specific dominant groups at the phylum and genus levels. There were differences in the gene functions of ginseng root-related bacterial communities between forestland and farmland. There were significant differences in the abundance distribution of rhizosphere bacteria among the different genotypes at the phylum and genus levels.

**Conclusions:**

There is a close relationship between the ecological environment and bacterial population structure, and the ecological environment of forestland is more conducive to the formation of rich rhizosphere bacterial populations; additionally, the genetic diversity is richer than that of farmland. The rhizosphere bacterial community structure of ginseng was influenced by genotype, and there was a correlation between the distance between ginseng genotypes and the stratified clustering of its rhizosphere bacterial community structure.

## Background

Ginseng, a perennial plant belonging to the *Panax* genus of the Araliaceae family, has various pharmacological effects, such as anti-tumor, immune regulator, anti-asthmatic, anti-depression, anti-fatigue, anti-viral, anti-oxidants, and anti-ulcer [1]. In the early twentieth century, the main method of ginseng cultivation in China was deforestation, which led to a serious destruction of forestland and loss of natural resources. Cultivated ginseng in farmland is a sustainable ginseng cultivation system and the leading model for ginseng planting industry development in China for the future. However, the low content of organic matter, poor physical condition of soil, serious diseases, long-term fertilization, and pesticides result in many problems for the planting of ginseng in farmland. Soil microorganisms can inhibit soil-borne diseases and promote plant growth [2]. Therefore, changes in bacterial and fungal communities in the rhizosphere soil during crop planting have attracted extensive attention [3]. The plant root system and surrounding soil environment, referred to as the rhizosphere, harbor a diverse and dynamic microbial community that directly contacts the root system and influences its physiological activity [4]. The emergence of high-throughput sequencing technology has provided an effective platform for exploring the structure and function of rhizosphere microbial communities [5]. Fang et al. [6] analyzed microbial communities in the rhizosphere soil of three types of ginseng by high-throughput sequencing. Compared to cultivated ginseng, the rhizosphere soil of wild ginseng has higher bacterial diversity and lower fungal diversity. Furthermore, the relative abundances of *Chloroflexi*, *Fusarium*, and *Alternaria* were higher in farmland-cultivated ginseng than in wild ginseng and understory wild ginseng. The results showed that the composition and diversity of the rhizosphere microbial communities were significantly different among the three types of ginseng. Soil microbial diversity and function in farmland were significantly lower than those in deforested land, and were affected by ginseng planting years. The abundance of common soil-borne pathogens of ginseng increased with cultivation years, leading to an imbalance in the microbial community [7]. Lei et al. [8] analyzed the abundance and diversity of endophytic bacteria in ginseng roots under three different cultivation modes: mountain-cultivated ginseng (MCG),field-cultivated ginseng (FCG)and deforestation with subsequent ginseng planting (DSGP). However, the effects of ginseng ecology and genotype on microbial community structure and diversity have not yet been reported. In this study, Chinese ginseng varieties in different environments and different ginseng varieties in the same environment were used to compare and analyze the effects of ginseng growth environment and genotype on microbial community structure and diversity, and to explore the relationship between bacterial community composition and environmental factors, the relationship between genotype and microbial community, and the function of ginseng rhizosphere bacterial genes in different environments. The objective of this study is to guide the cultivation of ginseng in farmland and greenhouse by taking the ginseng cultivated in forest land as a demonstration, and to obtain the characteristics of varieties and resources that are beneficial to the cultivation of ginseng in non-forest land.

## Results

### Comparison of soil physical and chemical indices in different ecological environments

In order to confirm the difference of soil environment, to provide the basis for the subsequent analysis of rhizosphere microbial population in different environments. The findings indicated that the main physicochemical properties differ significantly between forestland and farmland soils and that the same greenhouse-collected soils differ from each other in their physicochemical properties owing to different original soil sources, without significant changes in soil physicochemical properties owing to short-term environmental changes (Table 1).

**Table 1:** The determination results of soil physical and chemical properties of four ginseng planting sites

Sample	Organic matter(g/kg)	Available phosphorus (mg/kg)	Available potassium (mg/kg)	Total nitrogen(g/kg)	Total phosphorus (g/kg)	pH	Water content(%)	Altitude(m)
Forestland	48.07a	10.66c	331a	2.45b	0.17a	5.71a	10.75b	792a
Farmland	23.36c	28.04b	195c	2.34b	0.19a	5.65ab	9.90b	482b
Greenhouse S	44.53b	65.43a	259b	2.45b	0.20a	5.59ab	14.50a	225c
Greenhouse Z	48.77a	3.43d	339a	2.73a	0.24a	5.48b	7.56c	225c

### Correlation between soil factors and community structure of rhizosphere symbiotic bacteria of ginseng

There are substantial differences in environmental factors such as organic matter, available phosphorus, and altitude among soils in different ecological environments. The abundance of rhizosphere symbiotic bacteria Proteobacteria, Acidobacteria, Actinobacteria, Firmicutes, Gemmatimonadetes, and Cyanobacteria were significantly affected by soil factors such as soil total phosphorus, pH value, total nitrogen, organic matter, and rapidly available potassium. Among these, soil total phosphorus and pH were the most important factors affecting the level of the ginseng bacterial phylum community structure (Table 2). The abundance of the ginseng rhizosphere bacteria *Methylotenera, Chitinophaga, Bacillus, Nocardioides, Inquilinus* and *Mesorhizobium* was significantly affected by soil organic matter content. *Rhizobium* and *Agrobacterium* were closely related to soil available phosphorus. *Chitinophaga* and *Streptomyces* were significantly affected by soil available K. *Luteimonas* and *Solibacter* were significantly affected by soil total nitrogen. *Chitinophaga* and *Streptomyces* were significantly affected by soil available K. The genera affected by altitude included *Burkholderia* and *Luteibacter*, where soil organic matter was the most important factor affecting the horizontal community structure of the ginseng bacterial genera. Soil organic matter was the most important factor affecting the horizontal community structure of ginseng bacteria (Table 3).

**Table 2:** Pearson correlation between rhizosphere bacteria (class) and soil parameters in different environments

Phylum	Organic matter	Available phosphorus	Available potassium	Total nitrogen	Total phosphorus	pH	Water content	Altitude
Proteobacteria	-0.255	0.306	-0.325	-0.861	-0.993^b^	0.972^a^	0.570	0.751
Acidobacteria	0.424	-0.522	0.556	0.952^a^	0.924	-0.890	-0.689	-0.551
Actinobacteria	-0.988^a^	-0.071	-0.863	-0.702	-0.347	0.404	-0.096	0.216
Gemmatimonadetes	0.127	-0.228	0.184	0.779	0.989^a^	-0.968^a^	-0.537	-0.800
Verrucomicrobia`	0.868	0.330	0.602	0.413	0.159	-0.255	0.490	-0.273
Bacteroidetes	0.365	0.802	-0.008	-0.262	-0.314	0.197	0.942	-0.127
Chloroflexi	0.142	-0.246	0.204	0.791	0.990^a^	-0.968^a^	0.549	-0.790
Planctomycetes	0.221	-0.263	0.279	0.836	0.996^b^	-0.977^a^	-0.543	-0.780
Nitrospirae	0.365	-0.599	0.538	0.928	0.896	-0.849	-0.764	-0.485
Firmicutes	-0.984^a^	0.162	-0.895	-0.776	-0.422	0.468	0.015	0.237
Cyanobacteria	0.448	0.482	-0.996^b^	-0.738	-0.259	0.266	0.250	-0.097
Armatimonadetes	0.356	-0.439	0.465	0.924	0.962^a^	-0.933	-0.649	-0.639

Note: “^a^” and “^b^” indicate significant difference at 0.05 and 0.01 level

**Table 3:** Pearson correlation between rhizosphere bacteria genus and soil parameters in different environments

Genus	Organic matter	Available phosphorus	Available potassium	Total nitrogen	Total phosphorus	pH	Water content	Altitude
*Stenotrophomonas*	0.367	0.397	0.483	0.684	0.178	0.703	0.008	0.901
*Sphingobium*	0.364	0.881	0.667	0.192	0.281	0.268	0.609	0.706
*Burkholderia*	0.100	0.546	0.146	0.719	0.374	0.786	0.249	-0.983^a^
*Pseudomonas*	0.379	0.387	0.177	0.734	0.422	0.662	0.732	0.506
*Methylotenera*	-0.984^a^	0.040	0.847	0.337	0.686	0.398	0.126	0.225
*Janthinobacterium*	0.754	0.137	0.574	0.723	0.789	0.787	0.072	0.708
*Luteimonas*	0.633	0.342	0.662	0.903	-0.984^a^	0.903	0.459	0.617
*Novosphingobium*	0.330	0.414	0.458	0.696	0.199	0.719	0.013	0.918
*Sphingomonas*	0.406	0.679	0.606	0.839	0.931	0.786	0.802	0.383
*Pantoea*	0.372	0.401	0.489	0.679	0.171	0.698	0.004	0.898
*Rhizobium*	0.085	0.951^a^	0.458	0.125	0.217	0.228	0.778	0.661
*Kaistobacter*	0.914	0.007	0.765	0.561	0.790	0.622	0.063	0.469
*Rhodoplanes*	0.016	0.547	0.220	0.879	0.743	0.815	0.814	0.524
*Phenylobacterium*	0.306	0.772	0.577	0.401	0.085	0.468	0.451	0.838
*Nocardioides*	-0.992^b^	0.100	0.878	0.333	0.703	0.387	0.076	0.186
*Agrobacterium*	0.160	0.999^b^	0.537	0.138	0.439	0.033	0.897	0.445
*Dokdonella*	0.557	0.838	0.813	0.089	0.426	0.147	0.553	0.598
*CandidatusSolibacter*	0.489	0.610	0.648	0.865	0.967^a^	0.825	0.726	0.440
*Rhodanobacter*	0.915	0.260	0.872	0.626	0.906	0.656	0.199	0.380
*Flavobacterium*	0.233	0.914	0.170	0.046	0.175	0.075	0.902	0.455
*Inquilinus*	0.986^a^	0.021	0.844	0.199	0.590	0.262	0.195	0.104
*Mesorhizobium*	0.996^b^	0.089	0.878	0.251	0.647	0.306	0.118	0.114
*Lysobacter*	0.803	0.629	0.936	0.044	0.511	0.062	0.311	0.456
*Streptomyces*	0.926	0.524	-0.992^b^	0.396	0.831	0.397	0.346	0.012
*Chitinophaga*	-0.972^a^	0.382	-0.977^a^	0.179	0.668	0.199	0.124	0.118
*Microbacterium*	0.822	0.172	0.620	0.611	0.735	0.684	0.166	0.616
*Pseudoxanthomonas*	0.176	0.929	0.226	0.015	0.165	0.133	0.877	0.526
*Luteibacter*	0.310	0.468	0.063	0.755	0.516	0.827	0.227	0.957^a^
*Caulobacter*	0.103	0.936	0.291	0.095	0.147	0.209	0.831	0.610
*Nitrospira*	0.386	0.526	0.526	0.929	0.939	0.891	0.705	0.556
*Bacillus*	-0.984^a^	0.007	0.835	0.267	0.629	0.331	0.182	0.176
*Paenibacillus*	0.876	0.052	0.714	0.608	0.791	0.671	0.076	0.542
*Bradyrhizobium*	0.577	0.240	0.402	0.606	0.206	0.538	0.610	0.482
*Methylibium*	0.076	0.092	0.087	0.980^a^	0.712	-0.972^a^	0.427	0.872
*Flavisolibacter*	0.050	0.880	0.389	0.582	0.652	0.483	0.990^a^	0.052
*Thermomonas*	-0.548	0.196	0.530	0.949	0.936	0.961^a^	0.370	0.750

Note: “^a^” and “^b^” indicate significant difference at 0.05 and 0.01 level

### Comparison of the structure and function of the symbiotic bacterial community in the rhizosphere of Chinese ginseng varieties in different ecological environments

**Fig. 1:**
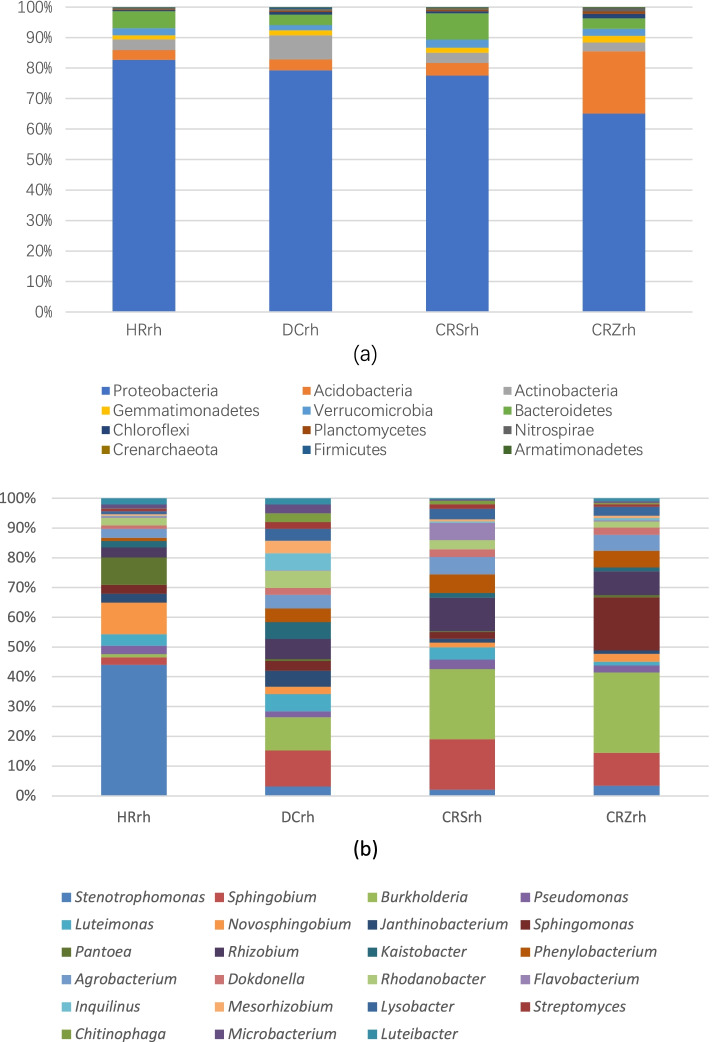
Distribution of rhizospheric bacteria in phylum level (**a**) and genus level (**b**) of Chinese ginseng in different environments

The common rhizosphere-dominant phyla of ginseng in different environments were Proteobacteria, Acidobacteria, Actinobacteria, and Bacteroidetes; however, the proportions of their respective rhizosphere-dominant phyla were different (Fig. 1a). The variation range of ginseng rhizosphere abundance of different dominant bacteria in different environments varies, including significant differences in rhizosphere bacterial abundance of Actinobacteria and Bacteroidetes in forestland and farmland environments.

There were differences in the dominant bacterial genera of Chinese ginseng rhizosphere bacteria in different environments, and different environments had their own dominant bacterial genera that were different from other environments (Fig. 1b).

The distribution of the dominant bacterial genera *Stenotrophomonas, Novosphingobium, Pantoea* in HRrh(rhizosphere soil of Chinese ginseng variety in Helong) was significantly different from that in other environments; the dominant bacterial genera *Burkholderia, Rhizobium, Sphingobium* in DCrh(rhizosphere soil of Chinese ginseng variety in Dunhua), CRSrh(rhizosphere soil of Chinese ginseng variety in greenhouse soil S), and CRZrh(rhizosphere soil of Chinese ginseng variety in greenhouse soil Z) were significantly different from their distribution in HRrh, and the distribution of *Sphingomonas* in CRZrh was significantly different from that in other environments, indicating that the proportions of dominant phyla and genera in the ginseng rhizosphere of various groups in different environments were significantly different.

**Fig. 2:**
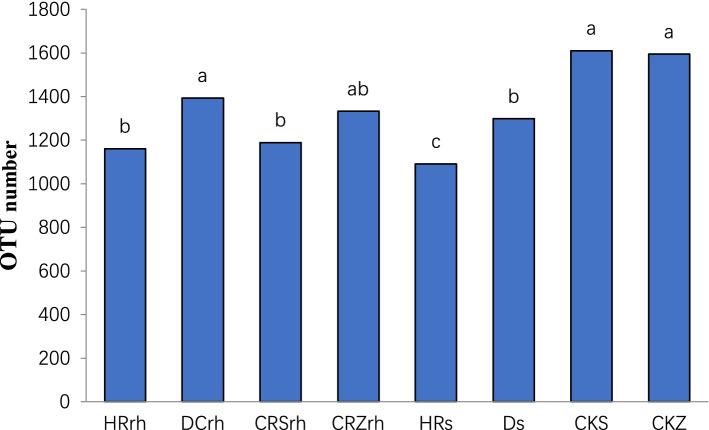
OTU number of root-related bacteria community for Chinese ginseng Damaya

The non-rhizosphere bacterial species were the least abundant in the forestland environment and differed significantly compared to the farmland and greenhouse environments, while the difference between the two samples in the greenhouse was not significant; the rhizosphere bacterial species were the least abundant in the forestland and differed significantly compared to the farmland environment (Fig. 2). Rhizosphere and non-rhizosphere bacterial species differed under different ecological conditions.

**Fig. 3:**
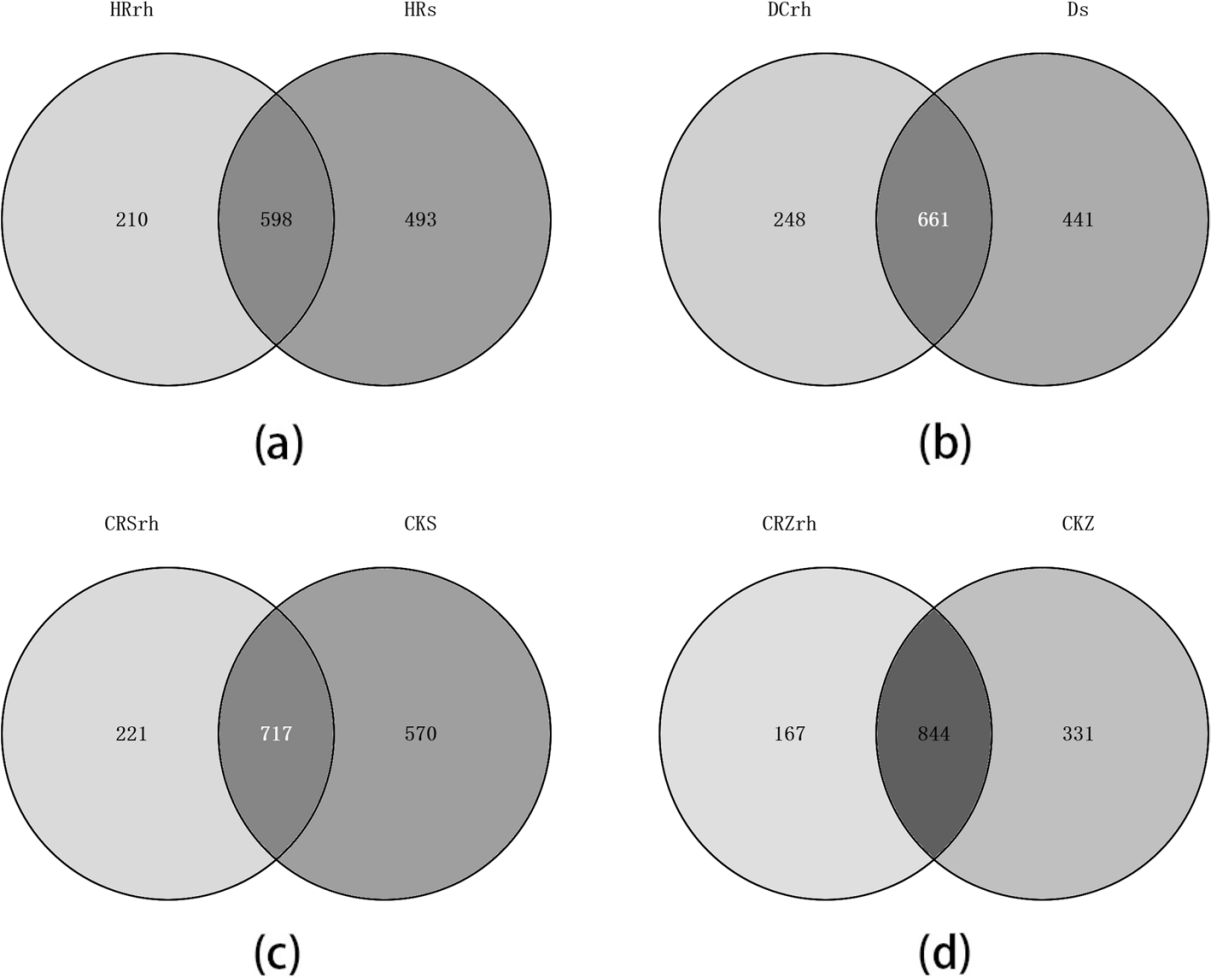
Venn diagram of rhizosphere and bulk soil bacteria of Chinese ginseng under different environments. **a** Forest; **b **Farmland; **c **Greenhouse S; **d **Greenhouse Z

The proportion of endemic bacteria enriched in DCrh was the largest and that enriched in CRZrh was the smallest. The proportion of endemic bacteria did not differ significantly among DCrh, HRrh, and CRSrh, but differed significantly from CRZrh (Fig. 3).

The bacterial population alpha diversity index includes Shannon, Simpson, Chao1, ACE, etc., and mainly focuses on species richness and uniformity in a local uniform ecological environment. The diversity and richness of HRrh rhizosphere bacteria were significantly different from those of DCrh, CRSrh, and CRZrh, whereas there were no significant differences between DCrh, CRSrh, and CRZrh. There were no significant differences between the diversity and richness of HRrh rhizosphere bacteria in the other environments (Table 4). This indicates that ecological differences have an effect on rhizosphere bacterial diversity and richness, with Chinese ginseng HRrh having the richest rhizosphere bacterial diversity.

**Table 4:** Alpha diversity index of Chinese ginseng community in different environment

Sample name	Shannon	Simpson	Chao1	ACE
HRrh	8.878a	0.925b	1393.294a	1409.215a
DCrh	7.246b	0.983a	1275.899b	1311.298b
CRSrh	7.124b	0.976a	1275.130b	1290.809b
CRZrh	7.879b	0.986a	1295.949b	1333.086b

**Fig. 4:**
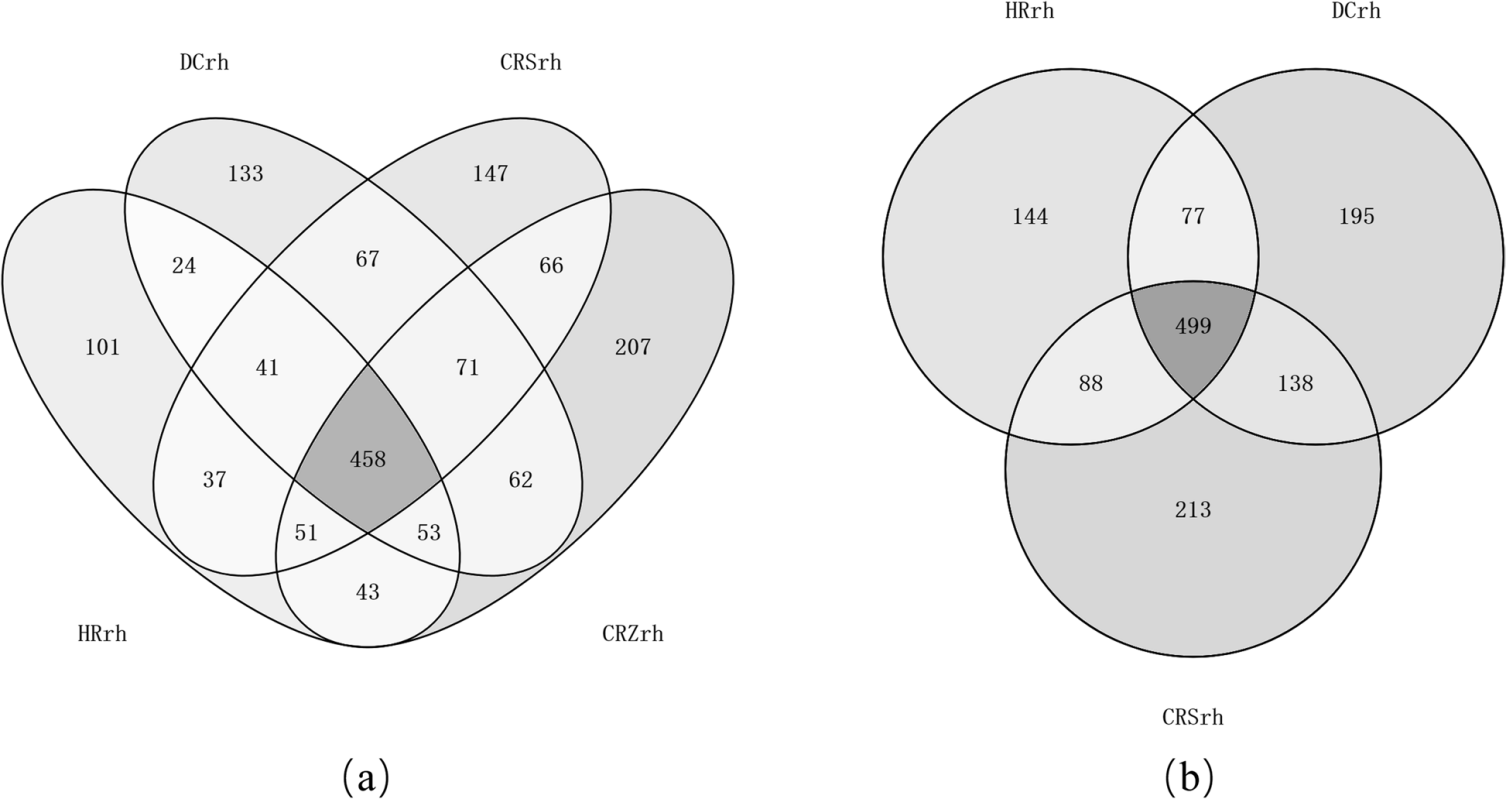
OTU distribution of soil bacteria in rhizosphere under different ecological environments. **a **Forest, Farmland, Greenhouse S, Greenhouse Z; **b **Forest, Farmland, Greenhouse S

There were 458 rhizosphere bacterial OTUs coexisting in the ginseng rhizosphere in all four environments, accounting for 12.49% of the total OTUs (Fig. 4a). As shown in Fig. 1b, the total abundance ratios of HRrh, DCrh, and CRSrh were all higher than 65%, whereas that of CRZrh was lower. Therefore, the OTU distribution of Chinese ginseng rhizosphere bacteria in the first three ecological environments was restored (Fig. 4b). A total of 1372 OTUs were obtained in the three ecological environments, and the number of OTUs in each ecological environment ranged from 808 to 956, with an average value of 891; 499 rhizosphere bacterial OTUs coexisted in the rhizosphere in the three ecological environments, accounting for 36.37% of the total OTUs. Thus, information on the Chinese ginseng core rhizosphere bacteria in different ecological environments was preliminarily obtained. The corresponding bacterial genera of these common OTUs included *Stenotrophomonas, Burkholderia, Sphingobium, Rhizobium, Pantoea, and Agrobacterium.*

Specific bacterial OTUs are present in the rhizosphere of Chinese ginseng in different ecological environments. Compared to farmland, the relative proportions of Bacteroidetes, Actinobacteria, Planctomycetes, and Gemmatimonadetes were higher in forestland ginseng. However, more strains of Proteobacteria, Acidobacteria, Verrucomicrobia, Chloroflexi, and Firmicutes were included in farmland ginseng-specific rhizosphere bacteria, and Nitrospirae and Cyanobacteria only existed in farmland rhizosphere-specific bacteria, but not in forestland. In addition, there was a higher proportion of unclassified phyla in forestland ecosystems.

**Fig. 5:**
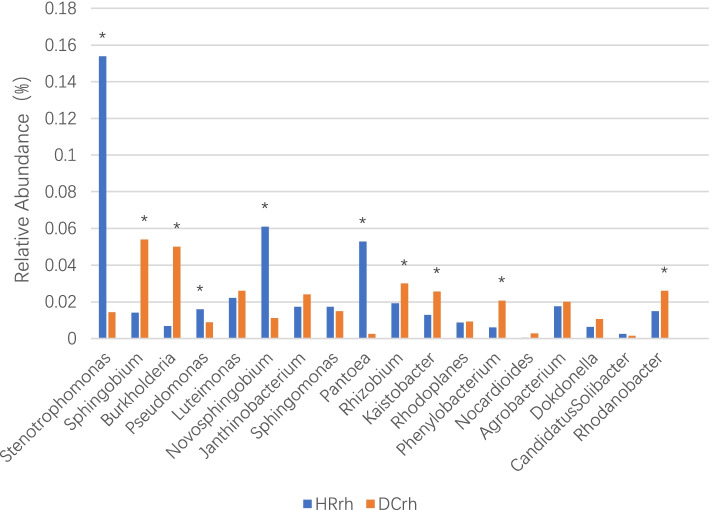
Genera distribution of diversity of rhizosphere bacteria between forestland and farmland

At the genus level, forestland-endemic bacterial genera included *Prosthecobacter*, *Cellulomonas*, *Sporocytophaga*, *Geobacter*, *Gemmatimonas*, *Hymenobacter*, *Bdellovibrio*, *Fimbriimonas*, and *Adhaeribacter*. Compared with farmland ginseng, the relative abundance of rhizosphere bacteria in forest ginseng was significantly higher, including *Stenotrophomonas*, *Novosphingobium*, *Pantoea* and *Pseudomonas*. Compared with the forest ginseng rhizosphere bacteria, the relative abundance of farmland ginseng rhizosphere bacteria was significantly higher in *Sphingobium*, *Burkholderia*, *Rhizobium*, *Kaistobacter*, *Phenylobacterium* and *Rhodanobacter* (Fig. 5). This indicated that there were significant differences in the composition of ginseng rhizosphere bacteria in forestland and farmland, and that the corresponding bacterial community was formed in a specific ecological environment.

### Comparison of ginseng bacterial gene function prediction under different ecological environment conditions

**Fig. 6:**
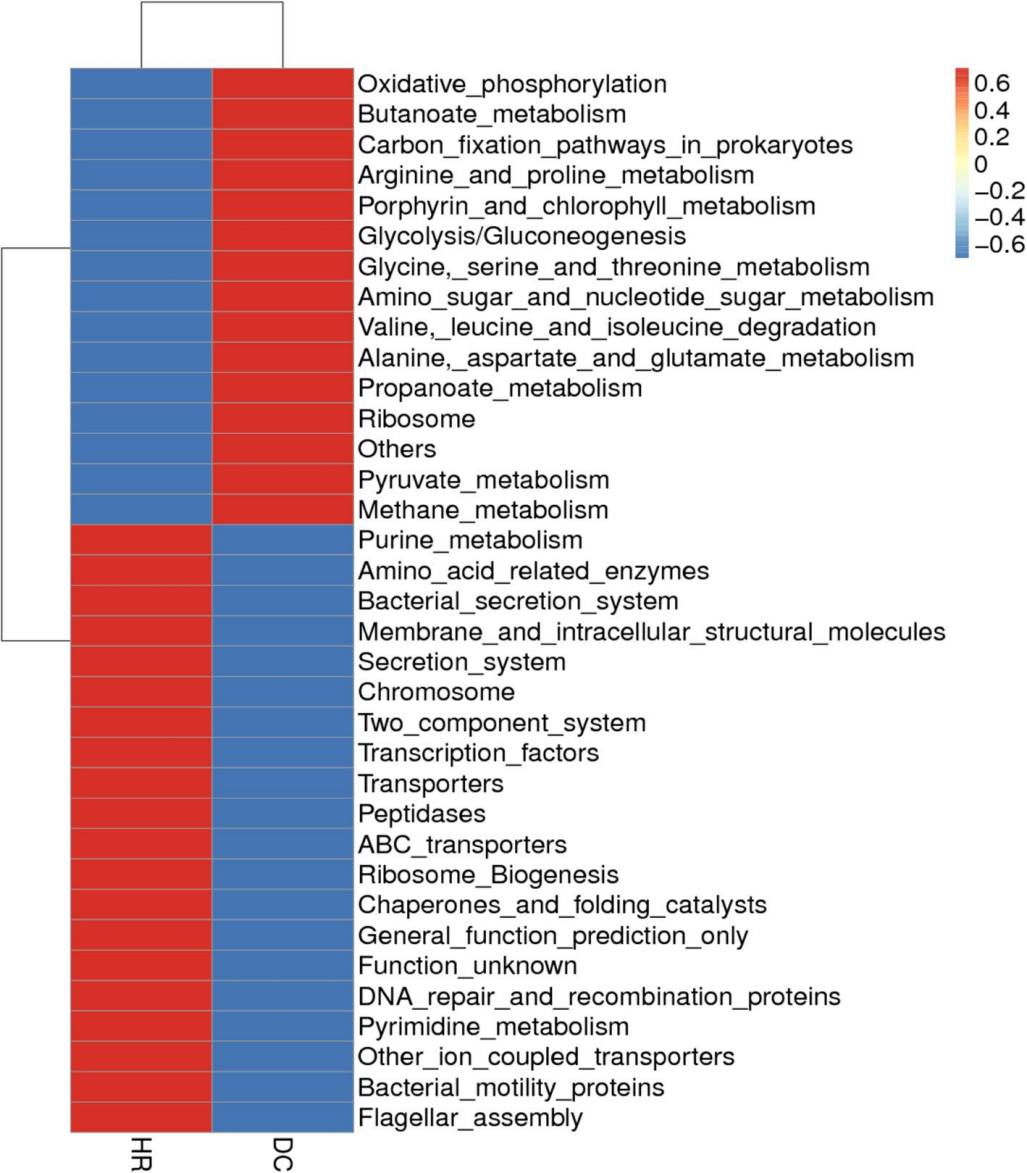
Clustering heatmap for PICRUSt function gene prediction of root related bacterial population from forest and farmland ginseng

As a phylogenetic marker gene, the 16S rRNA gene is a key tool for studying microbial communities; however, it cannot directly demonstrate the functional capacity of the community. PICRUSt was used to perform functional prediction based on the KEGG database for 16S rRNA sequencing data of root-related bacterial communities in woodland and farmland environments. A total of 5760 predicted functions were found, and the top 30 predicted functions can be seen in the heat map of the third classification level (Fig. 6).

According to the function prediction heat map, there were differences in the gene functions of ginseng root-related bacterial communities between forestland and farmland. Forestland ginseng root-associated bacterial communities had superior peptidases, chromosomes, molecular chaperones, folding catalysts, membrane and intracellular structural molecules, ribosome production, and bacterial motor proteins. In contrast, glycolysis/gluconeogenesis, pyruvate metabolism, glycolytic serine threonine metabolism, fatty acid metabolism, arginine and proline metabolism, propionate metabolism, valine leucine isoleucine degradation, and butyric acid metabolism were inferior to those of farmland ginseng. The above results indicate that the functions of the rhizosphere bacterial community of forest ginseng are more likely to be related to bacterial chemotaxis to root secretions, biofilm formation, and co-evolution with the host, whereas most of the functions of the rhizosphere bacterial community of farmland are related to nutrient metabolism, such as carbohydrates, amino acids, organic acids, and fatty acids.

### Comparison of community structure of symbiotic bacteria in the rhizosphere of ginseng of different genotypes

**Fig. 7:**
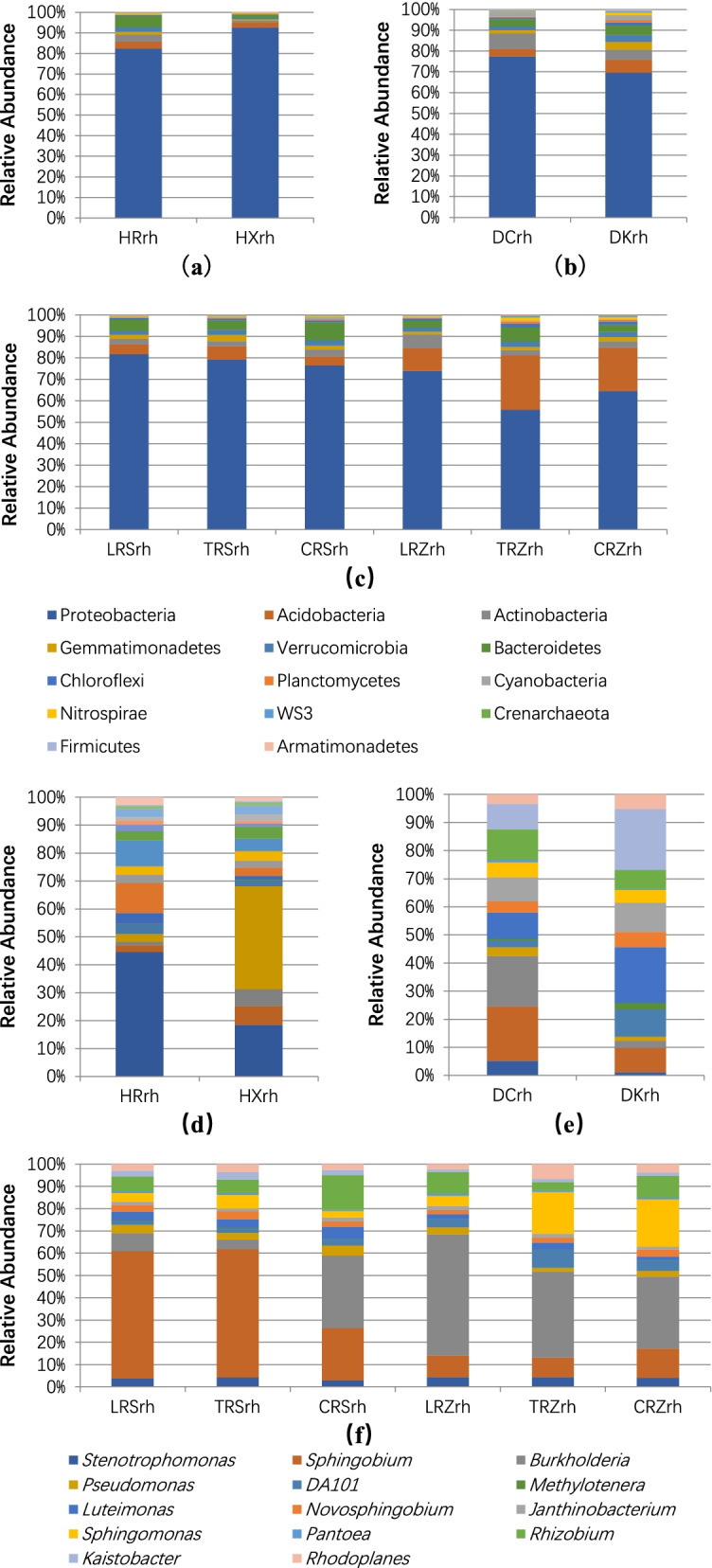
The relative abundance of the rhizosphere bacteria of the Chinese ginseng species Damaya and Western ginseng and the Korean ginseng species Lianfeng and Tianfeng for the test are shown at the phylum level (**a-c**) and genus level (**d-f**). **a**, **d** Forest; **b**, **e **Farmland; **c**, **f **Greenhouse S and Z

The range of variation in the abundance of the dominant phylum in the rhizosphere of ginseng of different genotypes also differed. Actinobacteria and Bacteroidetes showed significant differences in the rhizosphere bacterial abundance of HRrh and HXrh(rhizosphere soil of American Ginseng Varieties in Helong)(Fig. 7a), and there were significant differences in the abundance of Acidobacteria and Actinobacteria between DCrh and DKrh(rhizosphere soil of Korean Ginseng Varieties mixed line in Dunhua)(Fig. 7b). The comparison results of LRSrh(rhizosphere soil of Korean Ginseng Varieties Lianfeng in greenhouse soil S), TRSrh(rhizosphere soil of Korean Ginseng Varieties Tianfeng in greenhouse soil S), and CRSrh showed that Proteobacteria were significantly different between LRSrh and CRSrh, while the abundance of Actinobacteria and Acidobacteria in LRSrh was significantly different from that of other cultivars, and the abundance of Bacteroidetes in TRZrh(rhizosphere soil of Korean Ginseng Varieties Tianfeng in greenhouse soil Z) was significantly different from that of other cultivars (Fig. 7c). There were significant differences in the proportion of dominant genera in the ginseng rhizosphere among all groups in different environments and in the abundance of HRrh and HXrh at the genus level in *Stenotrophomonas, Pantoea and Pseudomonas* (Fig. 7d). The abundances of DCrh and DKrh in *Sphingobium, Burkholderia* and *Kaistobacter* were significantly different (Fig. 7e). The abundance of *Sphingobium* and *Burkholderia* in CRSrh was significantly different from that of other rhizosphere bacteria, and the abundance of *Burkholderia* and *Pseudomonas* in LRZrh(rhizosphere soil of Korean Ginseng Varieties Lianfeng in greenhouse soil Z) was significantly different from that of other rhizosphere bacteria (Fig. 7f).

These results indicated that there were significant differences in the abundance distribution of rhizosphere bacteria among different genotypes at specific phylum and genus levels, and genotypes had a great influence on the rhizosphere bacterial community structure of ginseng.

As shown in Table 5, differences in ginseng bacterial diversity among different genotypes in each habitat were compared horizontally. Shannon and Simpson indices reflected species diversity, while Chao1 and ACE indices reflected species richness; the results showed that, in the forest environment, the rhizosphere bacteria Shannon, Simpson, Chao1, and ACE indices of Chinese ginseng were significantly higher than those of American ginseng; in the farmland environment, the rhizosphere bacteria Shannon, Simpson, Chao1, and ACE indices of Korean ginseng varieties were significantly higher than those of Chinese ginseng Damaya. In the greenhouse environment, the Chao1 and ACE indices of the rhizosphere of Korean Tianfeng varieties were between those of Korean Lianfeng and Chinese varieties, and there was no significant difference in rhizosphere bacterial diversity between the Korean ginseng varieties Tianfeng, Lianfeng, and Chinese varieties. This indicated that the rhizosphere bacterial diversity of Chinese ginseng species was better than that of western ginseng, the rhizosphere bacterial diversity of Korean ginseng species was better than that of Chinese ginseng Damaya, and the rhizosphere bacterial richness of Korean ginseng Tianfeng was between that of Korean Lianfeng and Chinese ginseng species.

**Table 5:** Alpha diversity index of rhizosphere bacteria in different varieties of ginseng

Sample name	Shannon	Simpson	Chao1	ACE
HRrh	8.878*	0.925	1393.294*	1409.215*
HXrh	6.795	0.828	1223.456	1212.347
DCrh	7.246	0.983	1275.899	1311.298
DKrh	7.803*	0.988	1392.497*	1421.277*
LRSrh	7.148a	0.971a	1398.879a	1426.541a
TRSrh	7.533a	0.977a	1348.606a	1386.268ab
CRSrh	7.124a	0.976a	1275.130a	1290.809b
LRZrh	7.271a	0.968a	1135.124a	1185.967b
TRZrh	8.038a	0.991a	1196.259a	1224.556ab
CRZrh	7.879a	0.986a	1295.949a	1333.086a

* indicate significant difference at 0.05 level comparison between the two genotypes in the same environment

**Fig. 8:**
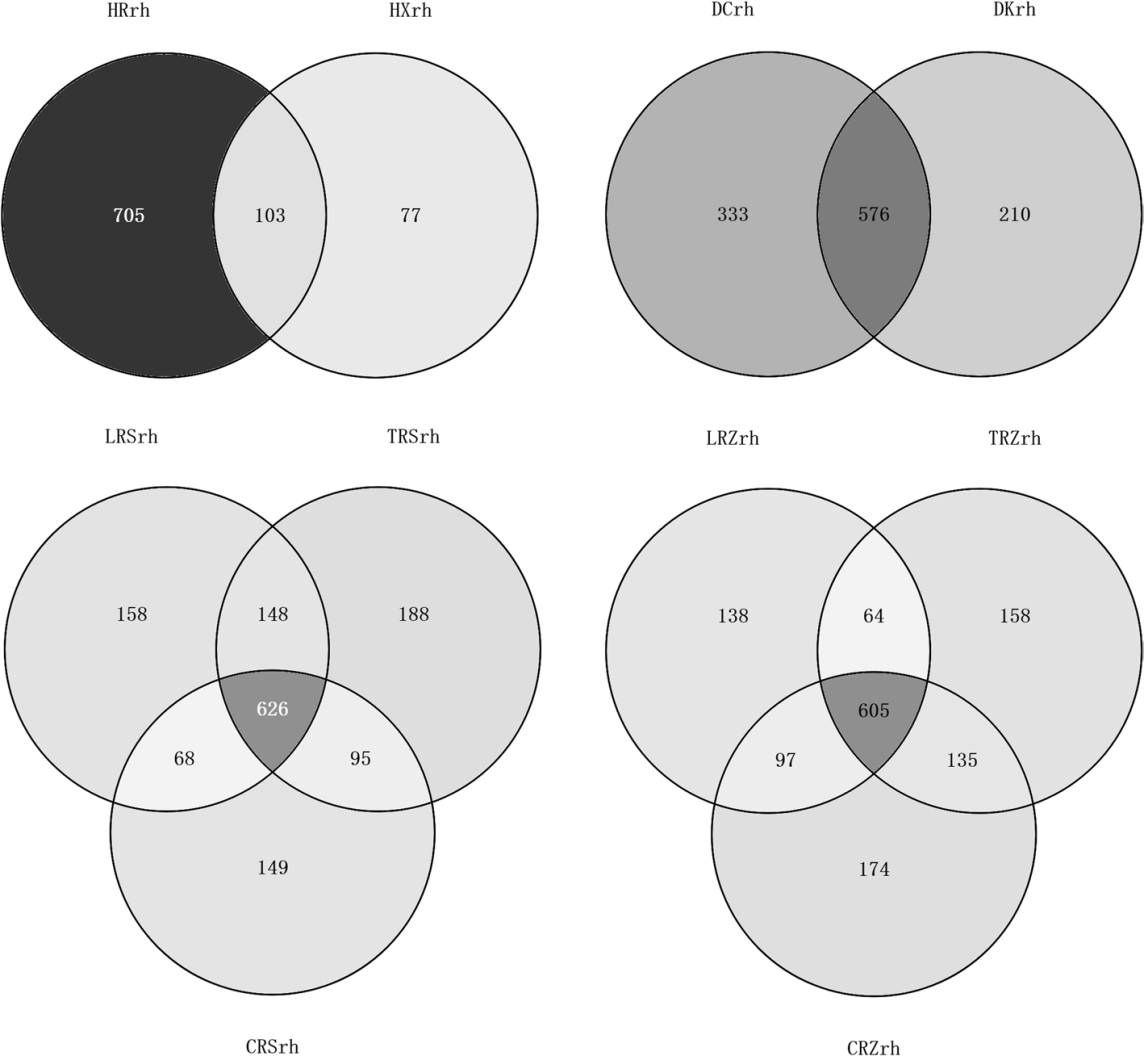
OTU distribution of rhizosphere bacteria in different varieties of ginseng, the calculated proportion of unique OTUs to all OTUs of the sample was used to indicate the specificity of species-enriched bacteria

The results showed that the proportion of unique OTUs to all OTUs was greater in Chinese ginseng than in Western ginseng, with significant differences in composition and structure. The proportion of OTUs common to both Chinese and Korean ginseng species was higher than 63%. The genera corresponding to these shared OTUs included *Novosphingobium, Pantoea, Rhizobium, Sphingomonas, Luteimonas, Rhodanobacter, Agrobacterium* (Fig. 8). The percentage of specific OTUs differed for each species. This indicates that differences in ginseng genotypes have a large effect on their rhizosphere bacterial species and distribution.

**Fig. 9:**
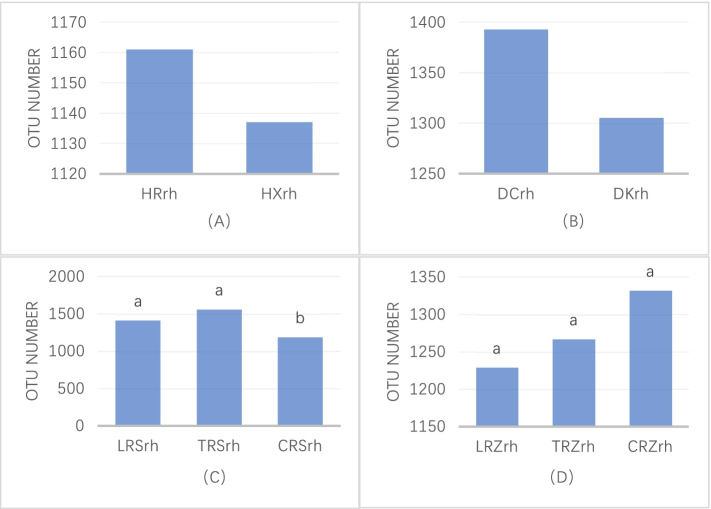
OTU number of rhizosphere bacteria in different varieties of ginseng

By comparing the differences in the number of bacterial OTUs of different varieties of ginseng in each habitat, the results showed that the number of bacterial OTUs in the rhizosphere of Chinese ginseng was greater than that of American ginseng (Fig. 9a) and Korean ginseng (Fig. 9b), but the difference was not significant; the number of rhizosphere bacteria of Korean ginseng Tianfeng > Korean ginseng Lianfeng > Chinese ginseng (Fig. 9c), and the differences among the three varieties were significant; the number of rhizobacterial OTUs was Chinese ginseng > Korean ginseng Tianfeng > Korean ginseng Lianfeng (Fig. 9d), but there was no significant difference among the three. This indicates that the number of rhizosphere bacterial OTUs between different varieties in the same environment may or may not be different.

**Fig. 10:**
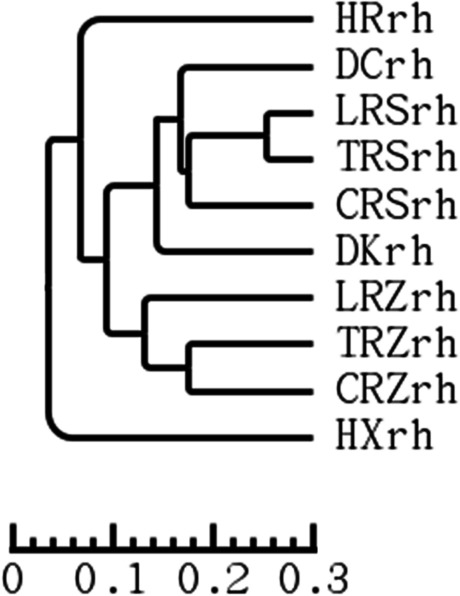
UPGMA clustering of all samples using weighted unifrac distances

The UPGMA clustering analysis based on weighted UniFrac distance (Fig. 10) could reflect the differences in the community structure of ginseng bacteria of different genotypes. Using 0.05 as the threshold can be divided mainly into HXrh and other genotypes, of which all the other genotypes are ginseng. At a threshold of 0.12, LRZrh, TRZrh, and CRZrh clustered together, TRZrh and CRZrh clustered together first and then with LRZrh, and the distance between them was not correlated with genotypes; at a threshold of 0.17, LRSrh, TRSrh, and CRZrh clustered together, where LRSrh and TRSrh clustered together first and with CRSrh clustered together and correlated with the distance between genotypes. The results showed that the distance between ginseng genotypes was correlated with the hierarchical cluster map of rhizosphere bacterial community structure.

## Discussion

### Relationship between ecological environment and the composition of rhizosphere symbiotic bacteria community

The yield and quality of ginseng are closely related to the conditions under which it is grown [9]. Xu et al. [10] improved the contents of organic matter, total nitrogen, and available phosphorus in ginseng through comprehensive improvement of farmland and found that it could promote the survival rate of ginseng seedlings and the growth of ginseng. According to the results of a comparative study on soil nutrients between forestland and farmland, Wang et al. [11] observed that the pH of the ground soil was the lowest, but the contents of ammonium nitrogen, available phosphorus, total nitrogen, total phosphorus, and organic matter were significantly higher than those of farmland and forest soil. Sun et al. [12] observed that the soil fertility of forest ginseng dominated by organic matter, total nitrogen, and total phosphorus was better than farmland ginseng. In the present study, there were significant differences in the main physical and chemical properties between forest and farmland soils, mainly reflected in the significant differences in the contents of organic matter, available phosphorus, and available potassium, while the differences in the contents of total nitrogen and total phosphorus were not significant, which may be related to the fertilization treatment of farmland sampling sites. In addition, soils collected in the same greenhouse have different physical and chemical properties due to different original soil sources, and the physical and chemical properties of the soil are not significantly changed in response to short-term environmental changes. Changes in the rhizosphere soil microbial community are related to crop species and soil type [13, 14]. Soil microbial diversity is important for maintaining the soil microbial community function [15]. Soil microorganisms are the most active part of the soil and are the driving force of soil material transformation and nutrient cycling [16]. In a comparative study of ginseng rhizosphere soil microbial communities in different environments, Song et al. [17] found that the microbial community of 4-year-old forest ginseng soil and farmland ginseng soil measured by the phospholipid fatty acid method was not significantly different. On the contrary, the results of the present study showed that there were significant differences in the bacterial composition of ginseng rhizosphere between forestland and farmland, and corresponding bacterial communities were formed in specific ecological environments. The diversity and richness of Chinese ginseng rhizosphere bacteria were affected by ecological differences, and the diversity of Chinese ginseng rhizosphere bacteria was the most abundant in forest environment. The differences in the results may be due to the different methods used to study the microbial community, as the phospholipid fatty acid method is often affected by soil physicochemical status and microbial growth environment [18]. The results of this study and previous studies showed that the contents of organic matter, total nitrogen, available nitrogen, and available phosphorus in the soil of field-cultivated ginseng were significantly different from those of farmland ginseng, and the different physical and chemical properties of the soil had a great impact on the soil microbial community. Therefore, significant differences in soil physicochemical indicators are bound to cause significant changes in soil microbial community structure and diversity.

With an increase in ginseng planting years, the richness indices of soil fungi, bacteria, and actinomycetes in the root region of ginseng decrease continuously, and the microbial population tends to be simple, which is not conducive to the growth of ginseng [19]. The results of the present study showed that the species of ginseng rhizosphere bacteria in the forest environment most suitable for the growth of ginseng was inferior than that in other ecological environments, indicating that the change in bacterial species is not the best indicator to describe the degree of soil health, and should be combined with the proportion of high-abundance communities and other factors for comprehensive analysis.

The results of the present study showed that ecological environment differences had a certain influenced the diversity and richness of rhizosphere bacteria, and the diversity of rhizosphere bacteria in Chinese forestland was the most abundant. There were significant differences in the proportions of rhizosphere bacteria and dominant bacterial genera in the different ecological environments. The difference in this bacterial population may be the reason for the growth of beneficial ginseng in forestland and the disease and yield reduction in farmland. Different soil conditions result in different microbial communities. On the one hand, the way plants shape rhizosphere microbes may lead to the collection of core microbiomes among plant common factors. However, certain plant-specific factors lead to the addition of microorganisms that are not members of the core microbial community. Root morphology and root exudates provide powerful evidence of microbiome formation by plant genetic factors [20]. Marginal cells with specific metabolism not only lead to obvious exudation of protein and mucus, but also produce low molecular weight compounds, which can be used as microbial nutrients or signal transduction substances [21].

The present study also revealed the presence of a relatively high abundance of *Stenotrophomonas, Novosphingobium*, and *Pantoea* in forestland environments, while farmland and greenhouse environments had a relatively high abundance of *Burkholderia, Rhizobium*, and *Sphingobium* in the proportion of rhizospheric bacteria, which were significant features of the rhizospheric bacterial populations of ginseng in different environments. Singh et al. [22] was the first to report that SBP-9, *Stenotrophomonas maltophilia*, has the potential to promote wheat growth under biotic and abiotic stresses directly or indirectly and can be further tested as a biocide for development at the field level. Moreover, this species is widely used in soil purification (bioremediation) because of its unique enzyme spectrum, which degrades various compounds [23]. From the above results, it can be seen that forest ginseng is different from the bacterial population of ginseng in farmland and greenhouse environments. Most of the groups with the highest proportion have the following functions: on the one hand, they have the ability to decompose difficult nutrients in plants; on the other hand, they produce bioactive substances that can inhibit pathogenic bacteria or reduce pollution. However, in this study, Bacteroidetes and *Stenotrophomonas* were not correlated with soil factors. Liao [24] found that cotton straw could significantly improve the abundance of Bacteroidetes, and separate application of straw and biomedical carbon could increase the abundance of *Flavobacterium* in Bacteroidetes. Therefore, in the process of planting ginseng in the field, we can try adding straw or biological carbon directly and introduce beneficial microbial agents, while monitoring the abundance dynamics of Bacteroidetes or *Stenotrophomonas* in the rhizosphere of ginseng to promote the evolutionary adaptation of beneficial bacteria to the farmland environment.

### Relationship between genotypes and the community composition of rhizosphere symbiotic bacteria

Plants regulate their rhizosphere microbiome in a host-dependent manner, with each plant species producing a specific microbiome [25]. As the phylogenetic distance increases, the differences in the rhizosphere microbial composition also increase [26]. In recent years, a large number of studies have confirmed that variety differences in the same crops are an important factor affecting rhizosphere microorganisms [27].

Jiang et al. [28] investigated the rhizosphere bacterial communities of 12 rabbiteye blueberry (RB) cultivars and demonstrated that the rhizosphere of the plant cultivar affects the bacterial association network. Huang et al. [29] showed that there were significant differences in rhizosphere microbial communities between the two *Brassica* species including *Bra*ssica *Sijiu* and *Brassica Cutai.* In a study on sweet potato rhizosphere bacteria, *Sphingobium, Pseudomonas, Acinetobacter, Stenotrophomonas,* and *Chryseobacterium* were found to be the differential genera of the low starch genotype compared with the two high starch genotypes [30]. Studies have shown that the soybean genotype significantly affects the rhizosphere bacterial community structure, and there are significant differences in the rhizosphere bacterial communities of soybeans with different genotypes [31]. Significant differences in the rhizosphere microbial community composition of wild and domesticated crops were found in bean [32] and maize [33].

The above research results indicate that the correlation between domestic and foreign plant varieties and the structure and function of specific bacterial populations is still unclear. Wang et al. [34] conducted a comprehensive investigation on rhizosphere soil bacterial and fungal communities of four ginseng varieties, CBGL (Korean ginseng), JYSH (common ginseng), SZSZ (Carnation ginseng), and TSBT (Pinata ginseng), and found that ginseng varieties are the main factors affecting the composition and diversity of the rhizosphere microbial community. In this study, the effects of genotype on rhizosphere bacteria were investigated in different ecological environments. There are great differences in the dominant genera between the two near-source species of ginseng and American ginseng in the forest environment, and the two varieties of ginseng in China and South Korea in the farmland environment. However, there are few differences in the dominant genera among the three varieties of ginseng in the greenhouse environment, and some of them are only quantitative differences. Based on this, we believe that the influence of ginseng genotypes on rhizosphere microbial populations is related to the ecological environment in which the samples are located and the genetic and developmental distance between genotypes.

The effects of plant genotypes on rhizosphere microbial composition were similar. Under the same experimental conditions, the rhizosphere microbial communities of Arabidopsis thaliana and Hordeum vulgare have specific taxonomic groups and different relative abundances [35]. There is a correlation between phylogenetic distance and rhizosphere microbial clustering of gramineous species, including rice variety [36] and Zea may [26]. However, the above conclusion is not applicable to Arabidopsis related species and ecotypes [37]. However, not all plant rhizosphere microorganisms are different from those in non-rhizosphere soils, and some species, such as maize and lotus [38], have accumulated a distinct rhizosphere microbial community, whereas others, such as Arabidopsis and rice, have accumulated rhizosphere microorganisms similar to those in non-rhizosphere soils. This also explains why the former shows a strong rhizosphere effect and the latter shows a weak rhizosphere effect. The results showed that there were significant differences in the abundance distribution of rhizosphere bacteria of different genotypes at the level of specific phyla and genera, and genotypes had a great impact on the community structure of the rhizosphere bacteria of ginseng. The number of rhizosphere bacterial OTUs may or may not differ among species in the same environment. There was a correlation between the distances between ginseng genotypes and the hierarchical clustering map of rhizosphere bacterial community structure. From the above results, it is clear that the selection and breeding of suitable varieties for farmland ginseng planting is crucial and necessary, considering the significant effect of genotype on the rhizosphere microbial community, which should be balanced with the construction of beneficial ginseng microbial communities. The results of this study provide a reference for the introduction of forest ginseng varieties in farmland and the breeding of farmland-specific varieties, suggesting that the restoration of beneficial ginseng microbial populations should be comprehensively analyzed in combination with local ecological factors such as climate and soil.

## Conclusions

The ecological environment of forest land is more conducive to the formation of rich rhizosphere bacterial community, and its genetic diversity is richer than that of farmland. The simpleness of bacterial species of rhizosphere in farmland depends on the characteristics of farmland soil, and the dominant genus are not the main factor leading to the different adaptability of ginseng in the forest and farmland. It is an important method to increase the genetic diversity of microorganisms for farmland ginseng. Soil organic matter, total phosphorus and pH are the most important factors affecting the bacterial community structure of ginseng. The rhizosphere bacterial community structure of ginseng was influenced by genotypes, and there was a correlation between the distance of ginseng genotypes and the stratified clustering of its rhizosphere bacterial community. The results lay the foundation for the selection and breeding of suitable varieties of ginseng for farmland cultivation and provide important theoretical and practical guidance for the development of ginseng bio-fertilizer and soil improvement of ginseng farmland.

## Methods

### Collection and processing of plant and soil samples

In October 2019, Chinese ginseng Damaya seeds (harvested from Helong Ginseng Farm, Yanbian Prefecture) and the seeds of Korean ginseng varieties Lianfeng and Tianfeng (provided by Dr. Hyunho Kim, Qingyang Experimental Station, Agricultural Research and Extension Service, Chungcheongnamdo, Korea) were selected. After surface disinfection, the seeds of the three varieties were sown into pre-prepared foam boxes containing two types of soil and placed in the ginseng greenhouse of the College of Agriculture, Yanbian University, without any treatment, such as pesticides and fertilizers before sowing and during the growth of ginseng, and were watered normally to ensure normal growth of ginseng.

In July 2021, ginseng samples from different ecological environments were collected, including (1) ginseng samples planted in the forestland (42.229 N/128.583E), and the varieties of Chinese ginseng (HRrh,HRs) and American ginseng (HXrh, HXs) collected in Helong Ginseng Field, Yanbian Korean Autonomous Prefecture, Jilin Province. (2) Ginseng samples planted in farmland (43.421 N/128.447E), and the collected varieties were Chinese ginseng (DCrh, Ds) and Korean ginseng (DKrh, Ds), located in the Dunhua Ginseng Field, Yanbian Korean Autonomous Prefecture, Jilin Province. (3) Greenhouse-planted (42.916 N/129.489E) ginseng samples were collected varieties included Chinese ginseng variety Damaya (CRSrh, CKS) and the Korean ginseng varieties Lianfeng (LRSrh, CKS) and Tianfeng (TRSrh, CKS), collected from forest soil pots on the campus of Yanbian University (S soil). The collected varieties were the Chinese ginseng variety Damaya (CRZrh, CKZ) and the Korean ginseng varieties Lianfeng (LRZrh, CKZ) and Tianfeng (TRZrh, CKZ), collected from Zhixin Forest Soil Pots (hereafter referred to as Z soil). The ginseng materials collected were all 2-year-old samples (RH: rhizosphere soil; s: CKS; CKZ: non-rhizosphere soil).

Five healthy ginseng plants were selected from forestland ginseng and farmland ginseng, and the roots, stems, and leaves of the whole ginseng plants were separated with sterile scissors. Additionally, In-situ soil 100 g were collected from each location and placed in sterile self-sealing bags and brought back to the laboratory immediately in ice boxes. The soil attached to the roots of ginseng was shaken off. The roots were put into a 50 ml sterilized centrifuge tube, add 40 ml phosphate buffer (pH 7.8), and shake the centrifuge tube in an air bath shaker 2 h at 200 rpm. Remove the ginseng root, centrifuge at 10,000 rpm. and discard the supernatant. The lower layer of the sediment was the rhizosphere soil sample. The soil samples collected from the sample plot were non-rhizosphere soils.

### Determination of soil physical and chemical properties

Soil water content was determined by the drying method, soil pH by the potentiometric method, soil organic matter by the volumetric method, soil available phosphorus by the Mo-Sb colorimetric method, available potassium by the flame photometer method, total nitrogen by the Kjeldahl method [39], total phosphorus by the HClO_4_-H_2_SO_4_ digestion method and elevation by the measuring instrument of MitutoyoLH-600E(Mitutoyo, Japan).

### DNA extraction, amplicon generation and Illumina Miseq sequencing

Bacterial genomic DNA was extracted using the FastDNA® SPIN Kit for Soil Kit (MP Biomedicals, Solon, USA). Genomic DNA was stored in collection tubes at -20℃ or -80℃ for subsequent experiments.

The diluted genomic DNA was used as a template for PCR amplification using specific primers 515F: GTG CCA GCM GCC GCG GTAA and 907R: CCG TCA ATT CCT TTG AGT TT with barcode in the 16S rRNA V4-V5 region [40]. The enzyme and buffer of Phusion® High-Fidelity PCR Master Mix with GC Buffer from Biolabs, UK were used, and the PCR amplification reaction system and procedure were as follows (30 μL): Phusion Master Mix (2 ×) 15 μL; Primer (2 μM) 3 μL; template DNA (1 ng/μL) 10 μL; sterile water 2 μL. Reaction procedure:98 °C pre-denaturation for 1 min; 98 °C for 10 s; 53 °C for 30 s; 72 °C for 30 s; total 29 cycles; 72 °C for 10 min. The PCR products were detected by electrophoresis on a 2% agarose gel.

Mixing and purification of PCR products: The samples were mixed at the same concentration as the PCR products. After mixing, the PCR products were purified by electrophoresis on a 1 × TAE 2% agarose gel, and the target band was recovered by gel cutting. The product purification kit used was the GeneJET Gel Recovery Kit (K0691 Thermo Scientific).

Library construction and sequencing runs: The libraries were constructed using the NEB Next® Ultra™ DNA Library Prep Kit for Illumina from New England Biolabs, and the constructed libraries were quantified and tested using Qubit, and then sequenced using the Illumina platform after passing the test.

### Data analysis

Species annotation and evaluation: Based on the UPARSE method of Edgar et al. [41], the quality sequences obtained were clustered into OTUs according to the principle of greater than or equal to 97% similarity, and the sequence with the largest number of each OTU was selected as the representative sequence, which is the species classification corresponding to the OTU. Based on the OTU clustering results, the representative sequences of each OTU were annotated to obtain the corresponding species information and abundance distribution. The diversity index of the samples was calculated based on OTUs, including Shannon, Simpson, Chao1, and ACE indices.

For the analysis, the method of random sampling of the majorizing sequence was adopted and a dilution curve was constructed based on the number of sequences and the number of OTUs they could represent to obtain the sampling depth of the sample. The default was to divide the OTUs at a 97% similarity level and create dilution curves for each sample.

Species composition analysis: Venn diagram based on OTUs was used to obtain information on species abundance and homogeneity within simple samples, as well as common and unique OTUs among different samples.

Comparative analysis of samples: β-diversity of bacterial community structure was analyzed by principal component analysis (PCA) based on the unweighted UniFrac matrix, hierarchical clustering of bacterial communities of each sample was analyzed by UPGMA the Unweighted Pair Group Method with Arithmetic Mean (UPGMA), and the Mantel test was used to analyze the correlation between the overall bacterial community and environmental physicochemical parameters. The above analysis was performed using the vegan packet of R software (Version 3.3.2).

Significant differences between treatments were compared at the 5% level by SPSS 19.0 software, using significant difference and Duncan's range checks.

### PICRUSt gene function prediction analysis

Full-length 16S rRNA gene sequences of the measured microbial genomes were used to infer the gene function profiles of their common ancestors. Extrapolation of gene function profiles of other untested species from the Greengenes database to construct a predictive gene function profile of the full spectrum of bacterial domains. Finally, the composition of the sequenced microbial community was "mapped" to a database to predict its metabolic function of the microbial community.

The study protocol must comply with relevant institutional, national, and international guidelines and legislation.

## Data Availability

The datasets used and analyzed during the current study are available from the corresponding author on reasonable request.
